# Decomposing Self-Control: Individual Differences in Goal Pursuit Despite Interfering Aversion, Temptation, and Distraction

**DOI:** 10.3389/fpsyg.2016.00382

**Published:** 2016-04-18

**Authors:** Rosa Steimke, Christine Stelzel, Robert Gaschler, Marcus Rothkirch, Vera U. Ludwig, Lena M. Paschke, Ima Trempler, Norbert Kathmann, Thomas Goschke, Henrik Walter

**Affiliations:** ^1^Department of Psychiatry and Psychotherapy, Charité Universitätsmedizin BerlinBerlin, Germany; ^2^Department of Psychology, Technische Universität DresdenDresden, Germany; ^3^Berlin School of Mind and Brain, Humboldt-Universität zu BerlinBerlin, Germany; ^4^Department of Psychology, Humboldt-Universität zu BerlinBerlin, Germany; ^5^Department of Psychology, FernUniversität in HagenHagen, Germany

**Keywords:** eyetracking, visual attention, disgusting pictures, erotic pictures, self-control task, willpower

## Abstract

Self-control can be defined as the ability to exert control over ones impulses. Currently, most research in the area relies on self-report. Focusing on attentional control processes involved in self-control, we modified a spatial selective attentional cueing task to test three domains of self-control experimentally in one task using aversive, tempting, and neutral picture-distractors. The aims of the study were (1) to investigate individual differences in the susceptibility to aversive, tempting, and neutral distraction within one paradigm and (2) to test the association of these three self-control domains to conventional measures of self-control including self-report. The final sample consisted of 116 participants. The task required participants to identify target letters “E” or “F” presented at a cued target location while the distractors were presented. Behavioral and eyetracking data were obtained during the performance of the task. High task performance was encouraged via monetary incentives. In addition to the attentional self-control task, self-reported self-control was assessed and participants performed a color Stroop task, an unsolvable anagram task and a delay of gratification task using chocolate sweets. We found that aversion, temptation, and neutral distraction were associated with significantly increased error rates, reaction times and gaze pattern deviations. Overall task performance on our task correlated with self-reported self-control ability. Measures of aversion, temptation, and distraction showed moderate split-half reliability, but did not correlate with each other across participants. Additionally, participants who made a self-controlled decision in the delay of gratification task were less distracted by temptations in our task than participants who made an impulsive choice. Our individual differences analyses suggest that (1) the ability to endure aversion, resist temptations and ignore neutral distractions are independent of each other and (2) these three domains are related to other measures of self-control.

## Introduction

Imagine you have to clean the toilet in your student housing with 20 inhabitants. Or imagine you are dieting, but you are offered a delicious piece of chocolate cake. Or imagine you are working on your annual tax declaration while your children try to grab your attention. What you need in all these different situations is self-control. You need to focus your attention and hold on despite aversive events, temptations, or distractions in order to reach a goal that you have set yourself. Until now, self-control has been measured using different paradigms, all of which entail conflicts between impulses and goal-directed behavior (Ach, 2006; Duckworth and Kern, 2011; Goschke, 2012). Only few studies assessed the specific selective spatial attentional processes underlying self-controlled behavior (Mann and Ward, 2007; Kelley et al., 2013). When focusing on individual differences, current tasks correlate at best moderately with each other, making it difficult to decide which of them is the most valid measure for trait self-control. One reason for such low correlations might be that all these tasks involve different impulse domains that are supposed to distract participants from goal attainment (Kuhl and Goschke, 1994; Kuhl and Fuhrmann, 1998; Tsukayama and Duckworth, 2010). For example, in some self-control tasks, participants have to endure aversive events such as pain (Kanfer and Goldfoot, 1966) boredom (Muraven et al., 1998) or solving an unsolvable task (Baumeister et al., 1998). Other self-control measures involve the ability to resist a positive temptation in order to reach a long-term goal – for example, resisting eating one marshmallow in order to receive two marshmallows later (Rosati et al., 2007; Mischel et al., 2011; Crockett et al., 2013, Privitera et al., 2015). A third group of self-control measures comprises cognitive tasks such as the Stroop or Flanker paradigm, in which emotionally neutral distractors need to be ignored (Gailliot et al., 2007). Automatic reactions to these impulse domains might differ between participants irrespective of their self-control ability (Tsukayama and Duckworth, 2010). Likewise, there is evidence that processes involved in the ability to control attention in a goal-directed manner might depend on the impulse category (Reeck and Egner, 2011; Jiang and Egner, 2014), thus providing further variability, which might underlie the weak correlations between self-control tasks addressing different impulse domains. Unfortunately, these correlations between different self-control tasks were rarely assessed within the same sample using the same task. Accordingly, other task-specific or sample-related differences might contribute to the finding of low correlations between self-control tasks. The current study introduces a modified attentional cueing paradigm that is intended to measure control of selective spatial attention mechanisms underlying self-control abilities in several impulse domains within one and the same paradigm (i.e., tolerating aversive stimulation despite an avoidance impulse, resisting erotic temptation, and ignoring neutral distraction). Furthermore, we investigate self-control in the face of the three impulse types from an individual difference perspective.

### Self-Control: Definition and Background

The terms ‘self-control’ and ‘willpower’ are often used to describe the same phenomenon. Baumeister et al. (2007, 351) defined self-control as the “capacity for altering one’s own responses, especially to bring them into line with standards such as ideas, values, morals, and social expectations, and to support the pursuit of long-term goals.” Often a dual-system perspective is explicitly or implicitly used in order to describe self-control conflicts (Hofmann et al., 2009). The dual-system approach to self-control describes a “hot” impulsive system that reacts to stimuli in a direct or automatic manner and a “cool” reflective system with higher-level goal representations (Metcalfe and Mischel, 1999). When these two systems are in conflict, self-control needs to be exerted in order to reach high-level goals.

Self-control is an important prerequisite for successfully reaching long-term goals: people try to control themselves in situations involving conflicts between higher-level goals and immediate gratification several times per day (Hofmann et al., 2012).

Deficits in self-control may also be related to several psychiatric disorders (Tangney et al., 2004), such as addiction (Bechara, 2005; Buhringer et al., 2008), attention deficit hyperactivity disorder (Schweitzer and Sulzerazaroff, 1995), and obesity (Konttinen et al., 2009), highlighting the importance of self-control research.

### Individual Differences in Self-Control - Domain-Generality vs. Domain Specificity

In line with the idea of the dual-system approach it has been proposed that self-control is domain-general in the sense that the same resources and mechanisms are involved independent of the specific type of self-control conflict (Baumeister et al., 2007; cf. Kana et al., 2013, for the issue of domain generality of cognitive control). This idea is supported by the finding that children who could resist eating one marshmallow in order to get two marshmallows later have been shown to be more successful later on in other life domains in which success requires self-control (e.g., school performance, ability to cope with stressful situations, ability to resist drugs, Mischel et al., 2011). As a consequence, the strategy for measuring self-control has typically been to select any task that involves a conflict between a momentary impulse and a higher-level goal without taking into account that there may be different impulse categories requiring different control processes.

In contrast, other authors argue for domain specificity in self-control. On the one hand, this includes studies, which found rather low correlations between and within different types of task categories used for self-control assessment. On the other hand, studies that used one task category (e.g., executive tasks) provide evidence for separable domains defined by the content of the impulsive reaction and their neural representation.

Concerning low correlations between task categories, Duckworth and Kern (2011) found in their meta analysis consistently low correlations between self-control tasks. They examined three different kinds of self-control measures: executive function tasks, delay of gratification tasks, and self- or informant-report questionnaires. While self-control questionnaires correlated with each other, delay of gratification tasks exhibited very low correlations with other delay of gratification tasks, albeit still higher than correlations with executive tasks. Convergent validity between questionnaires and self-control tasks was overall low or even non-existent. The authors concluded that “self-control is a coherent but multidimensional construct best assessed using multiple methods” (Duckworth and Kern, 2011, p. 1). Further support for this idea comes from Reynolds et al. (2006), who observed low correlations between questionnaires and executive tasks of impulsivity. Impulsivity shows a negative correlation with self-control (Ludwig et al., 2013) and can therefore be seen as a counterpart.

Although self-report measures of self-control have been shown to have high external validity as they correlate with life achievements (Tangney et al., 2004), the use of self-report measures for studying individual differences might have some disadvantages. One reason is that self-control is a socially desirable personality characteristic and thus self-report measures of self-control might be particularly prone to social desirability effects, lack of insight, and room for interpretation of item text (Johnson, 1981; Furnham, 1990; Back and Egloff, 2009). Accordingly, experimental tasks assessing different components of self-control are at least equally important for the evaluation of individual differences in self-control and the degree of domain-specificity.

With respect to findings regarding domain specificity within one task category, Tsukayama and Duckworth (2010) provided evidence in the self-report category for the idea that people’s self-control success depends on the kind of impulses that need to be controlled, and that individuals react differently depending on their susceptibility to specific impulse domains (see also Tsukayama et al., 2012).

Here, we propose that at least three different kinds of impulses should be considered: appetitive, tempting impulses triggering an approach reaction, aversive impulses triggering an avoidance reaction, and neutral stimuli that introduce a cognitive conflict.

The distinction between appetitive and aversive motivational stimuli has already been made in the influential theory on motivational personality structure proposed by Gray (1970). The theory posits two basic motivational systems: the behavioral activation system, which is responsible for facilitating behavior and generating positive affect, and the behavioral inhibition system, which is responsible for inhibiting behavior and generating negative affect. According to this framework, individual differences in the sensitivity to reward or potential negative outcomes can be explained by differences in these two systems. Factor analysis of the Behavioral Inhibition System and Behavioral Activation System (BIS/BAS) scales developed by Carver and White (1994) revealed that the behavioral inhibition and the behavioral activation system indeed form two independent motivational forces (Jorm et al., 1998).

Further evidence for distinct mechanisms for control over temptations and control over aversions comes from several neuroscientific studies using experimental self-control paradigms. These suggest that control over temptations mainly involves a regulation of striatal reward regions by the prefrontal cortex (PFC), whereas control over emotions (with most studies focusing on negative emotions), involves a down-regulation of the amygdala by the PFC (Heatherton and Wagner, 2011). Mak et al. (2009) compared emotion regulation of positive and negative emotions. In addition to overlapping regions they observed that regulation of positive emotion was uniquely associated with activity in dorsolateral frontal regions and that regulation of negative emotions was uniquely associated with activity in regions in the left orbitofrontal gyrus, the left anterior cingulate gyrus, and the left superior frontal gyrus. Erk et al. (2007) conducted an fMRI study with positive and negative emotional distractors during working memory maintenance. They observed valence-specific activations in prefrontal control regions. Specifically, they found that individual differences in amygdala activity in reaction to negative pictures was negatively correlated with inferior PFC activity and individual differences in activity of the ventral striatum in reaction to positive pictures was negatively correlated with left superior PFC activity. Together, these studies indicate partially distinct processes involved in the regulation of positive and negative emotion, which may be seen as a facet of self-control.

Sometimes we get into conflicting situations, not because we are reacting emotionally, but because there is a neutral distraction or a cognitive conflict without emotional content (Schacht et al., 2010; Jiang and Egner, 2014). It should be noted that cognitive conflicts can trigger negative emotions even if the content of the conflict seems neutral (Fritz and Dreisbach, 2013). However, it has been suggested that the resolution of cognitive and affective conflict nonetheless relies on partially distinct neural circuitries (Etkin et al., 2006; Ochsner et al., 2009; Reeck and Egner, 2011; Reeck et al., 2012). This indicates that emotionally neutral distraction should also be considered when looking at individual differences in self-control and their underlying attentional mechanisms.

We thus propose that self-control can be exerted in the face of negative (aversion), positive (temptation), or neutral distractors, all of which are hindering the pursuit of higher order goals and that these form independent components of self-control (i.e., that individuals high vs. low in one of these abilities will not necessarily score higher on the other abilities). The fact that most current self-control tasks restrict themselves to one of the three domains may explain the low correlations between them.

### The Present Self-Control Task

Our self-control task requires participants to attend to a cued target location while disgusting, erotic, and neutral pictures are presented as distractors. Participants could gaze away from the target location for the first 750 ms without any costs. After this initial period, when not fixated on the cued target location, participants might miss the target thereby risking a potential monetary reward, which they could get when performing well. Disgusting and erotic pictures are used because these naturalistic stimuli are evolutionarily relevant (Stark et al., 2005; Oaten et al., 2009) and therefore are highly likely to evoke aversion and attraction, respectively, in many participants. Aversive pictures are presented at the target location (participants have to control an avoidance reaction); erotic and neutral pictures are presented at a location different from the target location (participants have to control an approach reaction). Many authors have previously used distractors from different emotional valence (e.g., Elliott et al., 2002; Buchner et al., 2004). However, this has not been related to individual differences in self-control ability.

An attentional control paradigm was chosen because we assume that the ability to control attention in the face of distractors is a central component process supporting self-controlled behavior. Duckworth and Kern (2011) showed that performance in attentional tasks exhibited comparatively high correlations with informant questionnaires among various self-control measures. Additionally, attentional tasks have been used before in order to measure self-control (Carretie et al., 2004; Field et al., 2007; Back and Egloff, 2009). Furthermore, it has been shown that the acquisition of eyetracking data in attentional tasks can deliver additional information on individual differences in self-control (Kelley et al., 2013). For example, Friese et al. (2010) found that social drinking in combination with low working memory load affects initial orienting and attention maintenance on pictures of alcoholic beverages.

The aim of the present study was to assess the association of self-control and attentional control regarding three facets: ignoring aversive distractor, resisting tempting distractors and ignoring neutral distractors. We hypothesize that participants will not resist aversive, tempting, and neutral distractors at all times leading to costs in terms of error rates, reaction times (RTs) and increased gaze distance and variability from the target location. Furthermore, we expect that the three abilities form independent components of trait self-control. Moreover, we investigated the relationship of our task to conventional self-control measures. We therefore used an unsolvable anagram task in order to measure self-control during aversive events, a delay of gratification paradigm with food in order to measure self-control in the face of temptation, the Stroop task in order to measure self-control during neutral distractors, and questionnaires in order to assess general self-control ability.

## Materials and Methods

### Participants and General Procedure

The final sample consisted of 116 participants, who provided manual response data and 109 (51 male, mean age 25.89, *SD* = 3.80) who also provided eyetracking data. We tested 126 healthy current and former university students. Ten participants were excluded from behavioral analyses and an additional seven from eyetracking analyses (see Appendix A, Exclusion of Participants). Participants came for 3 days of testing and received 75 Euro for their participation.

On the 1st day of testing, participants performed an unsolvable anagram task, a color Stroop task, and a short test of fluid intelligence (Leistungsprüfsystem Unterteil 3, Horn, 1983). On the 2nd day, participants performed our self-control task and afterwards rated all pictures that were used in the task on valence, arousal, and attraction. On the 3rd day, the delay of gratification paradigm was conducted. During the 3 days, participants also performed additional tasks and completed a range of personality questionnaires not reported here. The study was approved by the local ethics committee of the Charité - Universitaetsmedizin Berlin, Campus Mitte. All participants gave writen informed consent in accordance with the Declaration of Helsinki.

### The Self-Control Eyetracking Task

**FIGURE 1:**
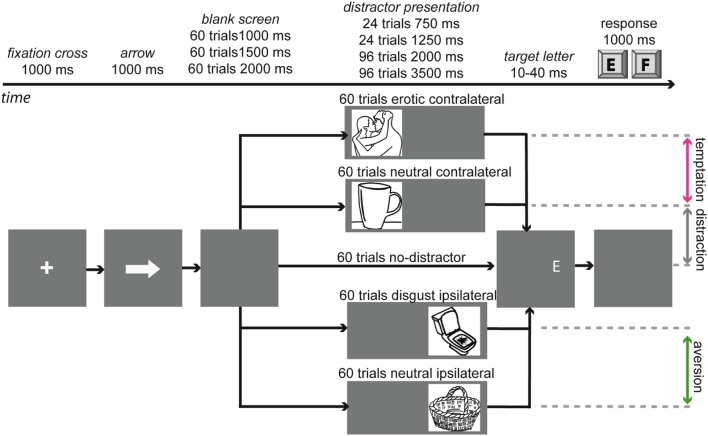
**Display of the trial Schema.** Each trial starts with a fixation cross followed by an arrow indicating the location of the next target letter 5.9° of visual angle left or right from the center. After the arrow presentation the screen was empty for a variable delay. Drawings are placeholders for photographs from the International Affective Picture System (Lang et al., 2008) and the internet.

Participants were instructed to indicate by button press whether they identified a white target letter as an “E” (right index finger) or an “F” (right middle finger). The target was briefly presented on a dark gray background. Participants were asked to respond as accurately and as quickly as possible. Four different types of distractors were possible: (1) a disgusting picture (e.g., of vomit, wounds, or spiders) at the location where the target letter would subsequently be presented, called the disgust condition; (2) a neutral picture presented at the target location, called the neutral ipsilateral condition; (3) a picture displaying a couple in an erotic situation displayed on the contralateral side of the screen relative to the target location, called the erotic condition; and (4) a neutral picture presented on the contralateral side of the screen, called the neutral contralateral condition (**Figure 1**). The letter size was 1.99° visual angle. The distractor pictures were 20.7° in width and 15.8° in height presented on a 36.5 cm by 27.5 cm screen with a resolution of 1024 pixels × 768 pixels.

Variable durations of distractors were introduced in order to prevent participants from anticipating the moment at which the target letter was presented. This required participants to fixate the target location continuously in order to ensure not missing the target letter. Prior to the first run, participants completed a training session that was excluded from the analysis. Participants were informed that after task completion, a lottery would take place, in which one trial would randomly be selected. If participants had responded correctly within 1000 ms on that trial, they received an additional 10 Euro (Paschke et al., 2015). Participants were informed that the money would be transferred to their bank account approximately 2 weeks after testing. The task was divided into four runs, each containing a calibration of the eyetracker followed by 75 trials. Trials were presented in a pseudorandom order. Picture presentation duration, letter, location of target presentation and transitions between conditions were balanced across the different distractor types. Directly after the picture presentation, the target letter was presented for 10 ms (if there was either no distractor or a distractor on the contralateral side of the screen), or for 40 ms if the picture was presented on the same side as the target. Pilot testing had indicated that this arrangement of presentation durations increases the probability that participants can report the letter only if they fixate the target position at the moment the target appears. Participants had 1000 ms for indicating the perceived letter by a key-press.

Stimuli consisted of pictures from the International Affective Picture System (Lang et al., 2008) and additional pictures from the internet. Prior to the study, all pictures were rated by 96 independent participants with respect to valence, arousal, and attraction/repulsiveness. The most appealing erotic pictures, the most repulsive disgusting pictures, and the most neutral pictures were selected and matched for brightness and picture complexity (see Appendix B for mean picture ratings, brightness, complexity, and red, blue, and green hue). There were 240 images (60 erotic, 60 disgusting, 120 neutral). Each picture was presented only once within the task.

Eyetracking data were acquired using a video-based eyetracker (sampling rate: 250 Hz, spatial resolution: 0.05°, Cambridge Research Systems, UK). Participants were seated 36 cm from the screen with their chin and forehead touching a chin rest. The refresh rate of the monitor was 60 Hz. Note that, due to the monitor refresh rate, true image durations differ slightly from the ones reported in **Figure 1**.

### Questionnaires

Three self-control questionnaires were used: (1) the Brief Self Control Scale (Tangney et al., 2004), (2) the Self-Regulation Scale (Diehl et al., 2006), and (3) the reverse score of the Barratt Impulsiveness Scale-11 as a measure of the counterpart of self-control (Patton et al., 1995). Participants completed these online at home prior to the start of the experiment. Since these three questionnaires are highly correlated, we reduced these data to one general self-report score using factor analysis (see Results). As an estimate of reliability, Cronbach’s alpha was calculated.

### Unsolvable Anagram Task

A computer version of the unsolvable anagram task was used. Participants were provided with a letter sequence on the screen. The task was to find a word by sorting the letters (using all letters and each letter only once). There were four solvable anagrams of increasing complexity, followed by one unsolvable anagram (see Appendix D for the list of anagrams used). Participants wrote down the solved anagram on a piece of paper and then continued to the next anagram by a button press. Participants were told that they could continue to do the task as long as they liked and could call the experimenter whenever they wanted to stop. The task ended if participants (1) called the experimenter because they did not want to continue, (2) gave up on the unsolvable anagram by clicking the “next anagram” button, or (3) had tried to solve the unsolvable anagram for 15 min. The time until the participants gave up on the first anagram, for which they did not find a solution, was taken as the dependent measure of self-control (thus, the maximum time is 15 min). Because this experiment entails only one target trial, it is not possible to calculate internal consistency reliability.

### Delay of Gratification

After 2 h during which participants did not eat, they were asked which of five different types of small chocolate sweets they preferred. After making the choice the participants were seated in a laboratory room and were presented with a plate with the unpacked chocolate. Participants were told that they could either eat the chocolate right away or could get double the amount in 45 min. Participants were then left in the room to make a decision. After 5 min the experimenter came back. At that point the chocolate had either been consumed or it was taken away for providing double the amount later. After the choice participants answered a questionnaire on their choice behavior, which was based on Rosati et al. (2007, Appendix C). This procedure was chosen in order to minimize confounding effects that limit the interpretation of delay task using unhealthy snacks in adults such as dietary restraints (Rosati et al., 2007) or interpretation of timing parameters (McGuire and Kable, 2013). Because this experiment entails only one target trial, it is not possible to calculate internal consistency reliability.

### Stroop Task

In the Stroop task (e.g., De Houwer, 2003), color words were displayed in different ink colors. Participants indicated the color of the word by pressing a button with the index fingers of their left or right hand. Two colors were assigned to the right hand button and two colors where assigned to the left hand button. Trials were congruent (50%) if the color word was the same as the ink color it was displayed in. Trials were incongruent (50%) if the color word did not match the ink color it was displayed in. Trials started with a fixation cross presented for a variable duration (1100, 3100, or 5100 ms). Afterwards the color word was presented for 300 ms followed by a fixation cross presented for 600 ms, during which participants could react. The difference between incongruent and congruent items (i.e., the Stroop effect) was used as a measure for self-control during neutral distraction, with a big difference corresponding to low self-control. Note that the task additionally allows for the distinction between (a) merely semantically incongruent and (b) semantically plus response incongruent items. Because we were interested in the general Stroop effect, we did not differentiate between these types of incongruence. Split-half reliability was calculated by correlating the Stroop effect (incongruent minus congruent) of odd trials with even trials while controlling for the three conditions (congruent, semantically incongruent, semantically and response incongruent).

### Data Analysis of the Self-Control Task

#### Analysis of the Task Effects

The analysis of the task effects was performed in Matlab (MATLAB version 2012b, Natick, Massachusetts: The MathWorks Inc.). For each condition the mean RT of all correct trials and the percentage of errors were calculated. In order to evaluate participants’ gaze path during distraction, mean gaze distance from the center point of the target location was estimated across time bins of 100 ms starting at distractor onset for all on-screen measurements. For the duration of 750 and 1250 ms the final bin contained only 50 ms. Additionally, in order to compare the different conditions, the means and the standard deviation of the 100 ms time bins were averaged across the entire duration. The standard deviation of the gaze distance was included as a measure of exploration behavior (i.e., how much participants looked around during the presentation of distractors). For the no-distractor condition, eyetracking recordings started 100 ms prior to target presentation during the delay between arrow and target. Eye gaze location during these 100 ms was taken as control condition for the entire period of the neutral distractor picture presentation (up to 3500 ms). This was done because participants’ gaze location in the final 100 ms prior to target onset should best reflect gaze location while expecting the target to occur. In order to evaluate the influence of aversive events, the disgust condition was compared to the neutral ipsilateral condition. The influence of temptation was estimated by comparing the erotic to the neutral contralateral condition. Neutral distraction was estimated by comparing the neutral distraction condition with the no-distractor condition.

#### Analysis of Correlations within the Task and with Other Self-Control Measures

All following data analysis steps were performed using SPSS (IBM SPSS Statistics 19). For the correlation analyses we used mean RTs (not the error data), because the distribution of the error rates was skewed with many participants making relatively few errors. Therefore, RTs can be assumed to be more sensitive in measuring individual differences in self-control. Three RT scores were calculated: aversion (disgust minus neutral ipsilateral), temptation (erotic minus neutral contralateral), and neutral distraction (neutral contralateral minus no-distractor condition). In order to relate task performance to questionnaire responses, a total self-control task score was also calculated by calculating the mean RT scores for aversion, temptation, and neutral distraction and then multiplying this by minus one. The multiplication by minus one was performed because the sum score of aversion, temptation, and neutral distraction refers to the degree of loss of control and not to self-control. In order to compare our task directly to other measures of self-control, we multiplied the “loss of control” score by minus one. Thus, the following formula was used for calculating the total score:

$$Total\,self-control\,task\,score=-1*\left(\left\{\left[RT_{disgust}-RT_{neutral\_ipsilateral}\right]+\left[RT_{erotic}-RT_{neutral\_contralateral}\right]+\left[RT_{neutral\_contralateral}-RT_{no\_distractor\_condition}\right]\right\}/3\right)$$

For all correlation analyses Pearson correlations are reported. For within-task correlations, where we claim that there is *no* association between measures, we additionally report attenuation adjusted correlation coefficients. Attenuation adjusted correlations provide an estimate of the strength of a correlation under the premise that there would be no measurement error. The rationale behind attenuating is that null results may be the consequence of measurement errors instead of being the consequence of orthogonality. Attenuation adjusted correlations were calculated by dividing the correlation of the two variables (*r*_xy_) by the square root of the multiplied reliabilities of the two correlated variables (*r*_xx_ and *r*_yy_; see Spearman, 1904 on attenuation adjusted correlation coefficients).

$$Attenuation\,adjusted\,correlations\,of\,\mathrm{var}iable\,x\,and\,y=\frac{\left(correlation\,of\,x\,and\,y\right)}{\left(square\,root\left(reliability\,x\,*reliability\,y\right)\right)}$$

Partial eta square ($\eta_{p}^{2}$) is reported as measure of effect size for significant group and condition comparisons.

#### Split-Half Reliability

For each measure of our self-control task, split-half reliability was calculated. For this, the pictures of each condition were divided into two halves. This was done by matching for distractor presentation duration and the response button but otherwise assigning pictures randomly into the two halves. From these, two difference-scores were calculated for each measure (aversion, temptation, and neutral distraction and a total self-control task score), as described above. Pearson’s correlation coefficients between the two versions of each measure were then calculated as estimates of reliability.

## Results

### General Results for the Conventional Self-Control Measures

#### Questionnaires

Because the three self-control questionnaires were highly correlated (all *r* > 0.5, all *p* < 0.001) and a factor analysis revealed that a single factor could explain 72 percent of the variance, they were reduced to this one factor using the regression method implemented in the SPSS factor analysis toolbox. Correlations between this factor and the Self-Regulation Scale, the Brief Self-Control Scale, and Barratt’s Impulsivity Scale were 0.86, 0.87, and -0.82, respectively. All three scales showed a high reliability in our sample: Self-Regulation Scale (α = 0.85), Brief Self-Control Scale (α = 0.83), and Barratt’s Impulsivity Scale (α = 0.82).

#### Unsolvable Anagram Task

Only 44 participants were able to solve all anagrams besides the unsolvable one. All other participants failed at an earlier anagram [anagram number (*n number of participan*ts): 1 *(13)*, 2 *(31)*, 3*(26)*, 4*(2)*]. The time in minutes until participants gave up on the first anagram that they were not able to solve was taken as the dependent variable (*M* = 5 min and 55.32 s, *SD* = 4 min and 23.32 s).

#### Delay of Gratification

For the analysis of this task, 31 participants were excluded because one of the three following criteria applied as measured by the post-experimental questionnaire: the participant indicated that they (1) were not hungry or had no appetite (2) did not like chocolate (3) were on a diet or were trying to control chocolate intake. Of the remaining participants, 24 participants chose to eat one piece of chocolate immediately and 61 chose to wait for two pieces of chocolate.

#### Stroop Task

Comparison of the congruent condition (*M* = 577.21, *SD* = 14.26) with the incongruent condition (*M* = 604.55, *SD* = 14.26) revealed a significant effect on RTs, *t*(115) = 2.712, *p* = 0.008. Reliability estimates revealed a reliability, *r*(114) = 0.41, *p* < 0.001.

### Main Task Effects: Comparison of the Different Conditions of the Self-Control Task

#### Reaction Times and Error Rates

**FIGURE 2:**
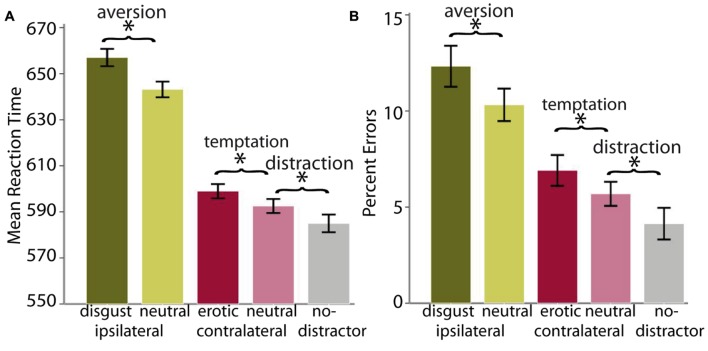
**Display of behavioral data.** **(A)** Mean RTs and **(B)** percent errors. Asterisks (^∗^) indicate a significant difference at *p* < 0.05, Bonferroni corrected for the six comparisons. Error bars represent the 95% confidence interval for within-subjects comparisons (Loftus and Masson, 1994).

Participants were generally slower and made more errors in the self-control conditions compared to their respective control conditions, indicating that additional processing was involved when tempting, aversive or distracting stimuli were present (**Figure 2**). As displayed in **Table 1**, all three *t*-tests yielded significant results for RTs and error rates (*p* < 0.05, Bonferroni corrected for the six comparisons). The exact values for mean ratings of valence, arousal, and attraction as well as RTs and error rates can be found in Appendix E. Note that the distribution of error rates was significantly skewed, with most participants committing few errors. Despite this, for simplicity, results of parametric analyses are reported here. However, non-parametric analyses of the error data yielded similar results (Appendix F).

**Table 1:** Results of within-subject *t*-tests of percent errors and reaction times (RTs).

Measure	Comparison	*M*(*SD*) difference	*t*-value	*p*-value	η^2^
Percent errors	Disgust vs. neutral ipsilateral	2.00 (5.82)	3.56	0.001	0.10
	Erotic vs. neutral contralateral	1.29 (4.81)	2.89	0.005	0.07
	Neutral contralateral vs. no-distractor	1.54 (4.45)	3.88	<0.001	0.12
Reaction times	Disgust vs. neutral ipsilateral	14.44 (24.53)	6.34	<0.001	0.26
	Erotic vs. neutral contralateral	6.83 (21.04)	3.49	0.001	0.10
	Neutral contralateral vs. no-distractor	7.55 (27.41)	2.88	0.005	0.07


*For all comparisons *df* = 115.*

#### Eye Tracking Data

**FIGURE 3:**
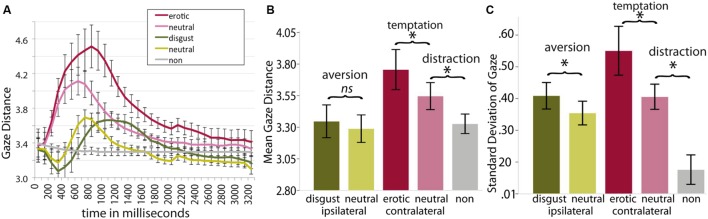
**Display of eyetracking data.** **(A)** the path of gaze distance in visual angle from the target during distractor presentation starting at distractor onset, **(B)** mean of gaze distance, **(C)** and of the standard deviation of gaze distance during the entire distractor presentation. Asterisks (^∗^) indicate a significant difference at *p* < 0.05, Bonferroni corrected for the six comparisons. Error bars represent the 95% confidence interval for within-subjects comparisons (Loftus and Masson, 1994).

Gaze path, mean gaze distance, and standard deviation of the gaze distance are displayed in **Figure 3**. The gaze path analysis revealed an effect of distraction for the different conditions starting 200 ms after distractor onset and peaking at around 1000 ms after distractor onset (**Figure 3A**). The conditions were compared over the whole distractor presentation period by calculating the means for aversion, temptation, and neutral distraction (see **Table 2** for the results of *t*-testing). There was a significantly higher gaze distance during the erotic condition in comparison to the neutral contralateral condition and during the neutral contralateral condition in comparison to the no-distractor condition (**Figure 3B**), but not for the disgust condition compared with neutral ipsilateral distraction. Additionally, aversion, temptation, and neutral distraction all resulted in a higher standard deviation of the gaze distance as compared to their respective control condition (**Figure 3C**; *p* < 0.05, Bonferroni corrected for the six comparisons, see Appendix E for exact values of all conditions). In order to calculate the precision of the eye tracking calibration the mean deviation from target dots during calibration was averaged across sessions and participants. The calculation revealed a mean deviation of 0.22° and a standard deviation of 0.01° visual angle, indicating high accuracy of eyetracking data.

**Table 2:** Results of within-subject *t*-tests of the mean gaze distance and the standard deviation of the gaze distance in degree of visual angle.

Measure	Comparison	*M(SD)* difference	*t*-value	*p*-value	*η^2^*
*M* gaze distance	Disgust vs. neutral ipsilateral	0.05 (0.44)	1.38	0.17	0.02
	Erotic vs. neutral contralateral	0.21 (0.58)	3.79	<0.001	0.11
	Neutral contralateral vs. no distractor	0.22 (0.80)	2.70	0.005	0.07
*SD* of gaze distance	Disgust vs. neutral ipsilateral	0.05 (0.20)	2.83	0.006	0.07
	Erotic vs. neutral contralateral	0.14 (0.39)	3.83	<0.001	0.12
	Neutral contralateral vs. no distractor	0.23 (0.38)	6.23	<0.001	0.26


*For all comparisons *df* = 109.*

#### Split–Half Reliability of all Reaction Time Task Measures

The reliability calculations revealed significant reliability for all task scores with a reliability of *r* = 0.44, *p* < 0.001, for the total task score. Furthermore, the three task sub-scores (aversion, temptation, and neutral distraction) showed a significant reliability (**Table 3**). Additionally to the reliabilities for the sub-scores, we calculated reliabilities for each condition (instead of using difference scores) as these are more comparable to questionnaire scores (which do not rely on difference scores either). This analysis revealed a reliability of *r* = 0.90, *p* < 0.001, for the erotic condition, of *r* = 0.89, *p* < 0.001, for the disgust condition of *r* = 0.90, *p* < 0.001, for the neutral contralateral condition, and of *r* = 0.74, *p* < 0.001, for the condition where no distractor is presented, indicating much higher reliability for single task scores than for difference-scores.

**Table 3:** Split-half correlations of self-control sub-scores (aversion, temptation, and distraction RT scores) reveal significant reliability and are displayed along the first diagonal of the table.

	Aversion	Temptation	Distraction
Aversion	*r*(114) = 0.24, *p* = 0.009^∗^		
Temptation	*r*(114) = 0.04, *p* = 0.67, a(r) = 0.14	*r*(114) = 0.30, *p* = 0.001^∗∗^	
Distraction	*r*(114) = -0.03, *p* = 0.72 a(r) = -0.04	*r* (114) < 0.01, *p* = 0.93 a(r) = -0.03	*r*(114) = 0.41, *p* < 0.001^∗∗^
Unsolvable anagrams	*r*(114) = 0.02, *p* = 0.81	*r*(114) < -0.01, *p* = 0.98	*r*(114) = -0.03, *p* = 0.72
	*i_r*(107) = -0.04, *p* = 0.67	*i_r*(107) = -0.02, *p* = 0.82	*i_r*(107) = -0.02, *p* = 0.85
Delay of gratification	*t*(83) = 1.51, *p* = 0.13	*t*(83) = 1.51, *p* = 0.13	*t*(83) = 0.53, *p* = 0.59
	*i_t*(76) = -1.32, *p* = 0.19	*i_t*(76) = 2.01, *p* = 0.048^∗^	*i_t*(76) = 0.31, *p* = 0.75
Stroop effect	*r*(114) = -0.12*, p* = 0.19	*r*(114) = 0.04*, p* = 0.66	*r*(114) = 0.09, *p* = 0.34
	*i_r*(107) = -0.04, *p* = 0.64	*i_r*(107) = -0.19, *p* = 0.048^∗^	*i_r*(107) = -0.08, *p* = 0.38


*Within-task correlation of the sub-score with each other reveals no significant correlation. Correlations attenuated for reliability are also reported [a(r)] but reveal correlations that are not much higher, suggesting independent components of self control. Correlations with the eyetracking scores (i_r) and t-testing with eyetracking scores (i_t) are additionally reported. Correlation across self-control measures using aversion (unsolvable anagrams), and distraction (Stroop) and t-testing of temptation (delay of gratification) reveal that eyetracking scores of temptation correlate positively with delay of gratification and negatively with the Stroop task RT effect while all other associations are insignificant. Asterisks (^∗^) indicate significant correlations at 0.05. Double asterisks (^∗∗^) indicate that correlations survive Bonferroni correction for multiple comparison (multiplication of p-value by total numbers of calculations reported in the table; in this case 15 number of comparisons) at 0.05.*

### Correlations within the Task and with Conventional Self-Control Measures

#### Within-Task Correlations of Reaction Time Effects between Aversion, Temptation, and Neutral Distraction for the Self-Control-Task

To assess if participants who were good at one of the self-control conditions were also good at the other conditions, we calculated within-task correlations of RT effects between the three self-control task measures. For the results see **Table 3**. Aversion and temptation were not correlated, neither were disgust reaction and neutral distraction. Temptation and neutral distraction were negatively correlated, *r*(114) = -0.31, *p* = 0.001. This negative correlation probably arose because these two variables are not independent: the neutral condition serves as the experimental condition in the neutral distraction comparison and as the control condition in the temptation comparison. In order to test this, we split the data set for the neutral condition and calculated two neutral contralateral conditions with every second trial in each condition. All even trials served as the control condition for temptation, and all odd trials served as the experimental condition for the neutral distraction comparison, revealing no significant correlation (see **Table 3**). The within-task correlations indicate that the three measures (aversion, temptation, distraction) measure different components of self-control.

#### Correlation of Self-Control Measures with Self-Reported Self-Control

**FIGURE 4:**
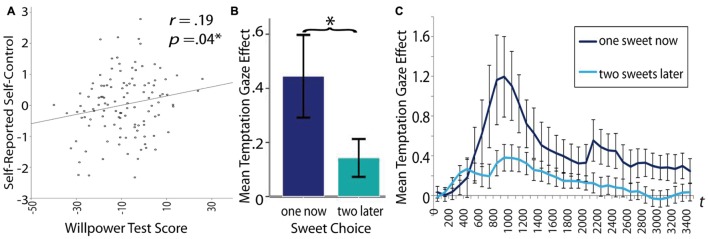
**Display of the association between different willpower measures.** **(A)** positive correlation between the total willpower task score and self-reported willpower, **(B)** the mean gaze distance temptation effect (distance from target letter in trials with erotic distractors minus distance in neutral trials) was significantly higher for participants who chose the small immediate reward than for those who chose to wait for the larger, but delayed reward (error bars represent 95% confidence intervals). **(C)** This association between choice in the delay of gratification task and distractibility by tempting pictures is displayed over the whole distractor period starting at distractor onset (error bars represent the standard error of the mean). Asterisks (^∗^) indicate a significant difference at *p* < 0.05.

Only the total score of our task correlated significantly with self-report (*r* = 0.19; **Figure 4A**), in contrast to the conventional tasks which did not show any significant relation (**Table 4**). The correlation of our task with self-report remained significant even when controlling for gender, intelligence, mean hours of sleep on the day of testing, and age, *r*(114) = 0.23, *p* = 0.013. Correlation of the three task sub-scales (aversion, temptations, and neutral distraction) with the questionnaire score revealed a correlation of temptation with self-reported self-control and no correlation of neutral distraction and aversion with self-reported self-control. This might indicate that the positive correlation of the total score with self-report may be mainly driven by the temptation sub-score (see **Table 3**). The results of the correlation analysis of the anagram task and the Stroop task with self-report and *t*-testing of participants who delayed gratification (*M* = -0.12, *SD* = 1.07, *n* = 24), in contrast to those who did not (*M* = 0.09, *SD* = 1.08, *n* = 62) revealed no relation between conventional self-control task and self-reported self-control. These results indicate that our task might more closely assess what is measured by self-control questionnaires compared to conventional tasks.

**Table 4:** Correlation of self-control tasks with self-reported self-control.

Task	Correlation with self-reported self-control
Self-control sum-score	*r*(114) = 0.19, *p* = 0.042^∗^
Aversion task score	*r*(114) = 0.02, *p* = 0.86
Temptation task score	*r*(114) = 0.19, *p* = 0.042^∗^
Distraction task score	*r*(114) = -0.11, *p* = 0.25
Unsolvable anagrams	*r*(114) = -0.06, *p* = 0.52
Stroop effect	*r*(114) = 0.05, *p* = 0.61
Delay of gratification	*t*(83) = -0.85, *p* = 0.40


*Our task shows a correlation with self-report in contrast to the conventional tasks, which do not show any relation. Only the temptation sub-score of our task shows a correlation with self-report, whereas disgust and distraction do not correlate with self-report. Stars (^∗^) indicate a significant correlation at 0.05. Note that none of the reported correlations would survive Bonferroni correction for multiple comparisons.*

#### Correlations between Domain-Specific Reaction Time Effects and Domain-Specific Conventional Self-Control Tests

There were no significant correlations between the domain-specific conventional self-control tests (anagrams, delay of gratification, and Stroop) and the respective domain-specific self-control RT scores (aversion, temptation, and neutral distraction; see **Table 4**). That is, there was no relation between the RT parameter of aversion and the time participants persisted with the unsolvable anagram task, no difference between participants choosing the small chocolate immediately (*M* = -12.67, *SD* = 18.28, *n* = 24) and participants waiting for two chocolates later (*M* = 5.28, *SD* = 20.91, *n* = 62), nor was there a relation between the parameter of neutral distraction and the Stroop incongruence effect.

#### Correlations between Domain-Specific Task Eyetracking Effects and Conventional Self-control Tests

Comparisons of the domain-specific impulse eyetracking parameters (aversion, temptation, and neutral distraction separately) with the respective self-control tasks revealed that the gaze distance scores in the temptation condition were associated with the external measure of temptation, while all other comparisons revealed no significant associations (see **Table 3**). Specifically, participants who chose to eat the small chocolate immediately showed a higher gaze distance to the target location during presentation of erotic stimuli (as compared to neutral stimuli; *M* = 0.44, *SD* = 0.74, *n* = 24) in comparison to participants who patiently waited for two pieces of chocolate later (*M* = 0.15, *SD* = 0.52, *n* = 54; **Figures 4B,C**), η^2^ = 0.05. Furthermore, correlating across domains revealed a significant negative correlation between the temptation gaze distance effect and the Stroop RT effect, but no relation for any other combination (**Table 3**). Note that the significant negative correlation between the temptation gaze effect and Stroop becomes insignificant when two possibly influential outliers are excluded from analysis (see Appendix G for the scatter plot and potential outliers).

## Discussion

In our study we put self-control to the test by using aversive, tempting, and neutral pictures in an eye gaze task. We show robust behavioral effects of aversion, temptation, and neutral distraction reflected in higher error rates and RTs as compared to control conditions. Gaze pattern analysis reveals higher gaze distance from the target location during presentation of erotic pictures as compared to neutral pictures (temptation), and for neutral pictures compared to the no-distractor condition (neutral distraction). Additionally, there was more looking around during aversion, temptation, and neutral distraction, reflected in a higher standard deviation of the gaze distance to the target. The eyetracking data thus indicate that the three distractor types indeed led to behavioral effects and not merely to arousal reactions. The total self-control task score obtained from the task (reflecting the ability to resist any of the three distractions) correlated with self-reported self-control. This indicates that our task measures a construct that is overlapping with self-report measures. Importantly, performance in the three self-control conditions did not correlate with each other, suggesting high individual differences in the effect of different emotions on information processing. This suggests that resisting pleasant temptations, enduring aversion, and ignoring neutral distractions are central and independent aspects of self-control.

### The Eye Gaze Task as a Measure of Self-Control

Our data indicate that our task can indeed measure individual differences in self-control, and that it might be better suited for this than conventional measures. The score obtained from our task correlated significantly with self-reported self-control. In contrast, neither the anagram task, nor the delay of gratification task, nor the Stroop task correlated with self-report in this study. This is in line with a meta-analysis performed by Duckworth and Kern (2011). They showed that many commonly used cognitive self-control tasks do not correlate with self-reported self-control or correlate only weakly. The reason for this absence of correlations might be that conventional tasks only capture a fraction of self-control (Duckworth and Kern, 2011). In contrast, the advantage of our task is that we focus on attentional control assumed to be an important component of self-control and that provide a measure of various components of attentional control within one task: enduring aversive distractors, resisting tempting distractors, and ignoring neutral distractors.

Interestingly, correlation of the task components (aversion, temptation, and neutral distraction) with the questionnaire score revealed that temptation by itself is correlated with self-reported self-control (*r* = 0.19), whereas aversion and neutral distraction are not. This might indicate that the questionnaires used in this study mostly assess individual differences in resisting temptations, but do not measure individual differences in standing aversions or ignoring neutral distractions. This further highlights the need for investigating different impulse types more closely, also on a questionnaire level.

Another advantage of our paradigm is that the impulse categories can easily be extended or exchanged depending on the impulse category of interest. This can be particularly beneficial when the aim is to study patient groups whose self-control changes in reaction to particular impulse categories, for instance in addiction or eating disorders. For example, it has been shown that addiction might lead to an increased capture of attention and automatic approach reactions regarding the addictive substance (Field et al., 2007; Friese et al., 2010; Wiers et al., 2013). Thus, to study addiction, our task could be used with pictures of the addictive substance as tempting distractors.

Despite these promising findings, our task should be further validated as a measure of self-control. One reason is that the correlation with self-report does not substantially exceed the correlations reported by Duckworth and Kern (2011) and does not survive Bonferroni correction for multiple comparison. Furthermore, a prediction of real life self-control on the basis of task results could be seen as more convincing evidence for construct validity than a correlation with self-report. The reason is that questionnaires, although having many advantages such as high internal consistency and reliability (Carver and White, 1994; Tangney et al., 2004; Diehl et al., 2006), still rely on subjective judgment of one’s own personality. This can be problematic, because correlations with external measures such as school performance may be spurious if the participants judge their own self-control based on their school success. The reason is that school success might not be solely related to self-control, but is profoundly influenced by other variables such as intelligence (e.g., Spinath et al., 2010), memory skills (Aronen et al., 2005), socioeconomic status (e.g., White, 1982) or self-efficacy believe (Zajacova et al., 2005). If participants judge themselves based on their school success, but school success is profoundly influenced by other factors, a positive correlation with self-report might not be meaningful for validation of the questionnaires.

Further validation in addition to questionnaires is therefore necessary. This could be accomplished by investigating participants that face a particular self-control challenge (e.g., dieters, Hare et al., 2009). The task design allows exchanging current stimulus material with pictures that may challenge self-control for that particular group (e.g., in the case of dieters, snack pictures). Another possibility for validation may be the use of experience sampling. Hofmann et al. (2012) used a beeper that samples the presence of self-control problems, the kind of problem and the executed behavior (controlled vs. not controlled) several times per day. On average, participants reported a self-control conflict every second time they were beeped. A correlation with these kinds of real-world self-control measures would be very convincing.

One could argue that a limitation of our task is that it refers to a relatively short time scale, as the reward for successful self-control is obtained already 2 weeks after testing. Moreover, the actual duration of active goal pursuit (i.e., the task itself) was only about 25 min. This relatively short time frame may also result in stronger effects of state factors that may influence self-control (e.g., blood glucose, Baumeister et al., 2007). In contrast, some (but certainly not all) real-life situations require the exertion of self-control over months or years (e.g., graduating, keeping a life-long healthy diet) and it was suggested that this long-term grit might be an even more essential key to life-success than relatively short-term self-control ability (Duckworth et al., 2007). Since some questionnaire items and temporal discounting items of conventional measures involve such potential long-term goals, this is one advantage of questionnaires and temporal discounting over our self-control task. However, there are also many real-life self-control situations in which the goal can be reached relatively soon, such as resisting eating the pralines that you bought as a gift or cleaning the toilet. Additionally, long-term goal pursuit can be decomposed into several smaller self-control situations. For example, graduating might be a long-term goal, but writing an essay might take just a few hours and can serve the relatively short-term sub-goal to pass one particular class. Therefore, we think that our task captures a wide range of everyday self-control problems.

A potential factor that might have resulted in a decrease of correlation coefficients in our study is the relatively homogenous group with all participants being university students or former students. The advantage of this homogeneous sample is a reduction of confounding factors. However, a more heterogeneous sample may result in higher variance of self-control scores and thereby a higher probability of detecting correlations.

The total task score of our task exhibited reliability similar to the reliability of the Stroop effect (our Task: *r* = 0.46, Stroop effect: *r* = 0.41) and similar to the reliability reported for Stroop in the literature (*r* = 0.46, Strauss et al., 2005). For emotional Stroop even lower reliabilities have been reported in previous studies (Strauss et al., 2005; Dresler et al., 2012) indicating that our task’s internal coherence is comparable to commonly used tasks. Additionally, single condition reliabilities reveal a much higher reliability (r between 0.75 and 0.90), indicating that reliabilities are lower when using difference scores than when using single task scores. It has to be kept in mind that in single score reliability estimates, confounding factors may boost reliability-measures because general response tendencies such as fast reacting or extreme responding that do not relate to the trait of interest can be consistently high or low in particular participants leading to exaggerated correlations in split-half estimates without increasing reliability for measuring the trait of interest.

The generalizability to populations of a different moral background or a homosexual population should be further investigated, as the current study does not allow us to draw any conclusions in that direction. See Appendix H for gender comparison (no significant difference for error rates, RTs and gaze distance, only for standard deviation of the gaze distance). Furthermore it should be noted that, although we matched pictures on brightness and complexity and the compared categories do not differ significantly on these dimensions, stimuli could be matched even more precisely regarding these and other dimension such as color, line properties and number of people in the image in future studies, as low level image properties have been shown to guide attention and might explain why participants were more distracted by erotic and disgusting pictures than their respective control stimuli.

### Resisting Temptations, Enduring Aversion, and Ignoring Neutral Distraction as Independent Aspects of Trait Self-control

The self-control sub-scores (aversion, temptation, and neutral distraction) seem to measure different aspects of self-control. This is reflected in the absence of correlations between these scores across participants. Thus, people who have a hard time suppressing approach reactions toward pleasant stimuli (e.g., resisting a cake during a diet) are not necessarily the ones who have a hard time suppressing avoidance reactions concerning unpleasant situations (e.g., going to the dentist). Distractibility by neutral distractors also did not correlate with distractibility by erotic pictures or by disgusting stimuli. This absence of correlations cannot be explained by an absence of reliability, as split-half correlations reveal moderate reliabilities for all three sub-scores. Thus, our findings indicate that resisting temptations, enduring aversion, and ignoring neutral distraction are independent aspects of self-control. Note, that temptation and distraction reliability even survive conservative Bonferroni correction for multiple comparisons, whereas aversion does not survive this correction and should therefore be interpreted with caution.

These results suggest that the impulsive system in the dual-system approach to self-control (Hofmann et al., 2009) should be subdivided (Heatherton and Wagner, 2011). Dual-system approaches typically assume that self-control challenges entail a conflict between an impulsive system and a reflective system (e.g., Epstein, 1990; Metcalfe and Mischel, 1999; Strack and Deutsch, 2004; Hofmann et al., 2012). Although we agree with the basic assumption of this model, our data imply that reality is more complex, and that at least the impulsive system consists of several sub-components. Our data indicate that one should differentiate impulses with respect to aversion, temptation, and distraction. We cannot, however, determine whether the three components are sufficient for completely explaining self-control behavior, or which other facets might be relevant. Future studies should investigate this.

Of note, our study attempted to decompose the impulsive part of the dual-system framework. We therefore cannot draw conclusions regarding the reflective system. This means that our data do not directly contradict Baumeister et al.’s (2007) hypothesis about the reflective system relying on a single, domain-general control resource. However, as there is also evidence for separable control processes depending on the content of attentional control, we consider it important to similarly investigate a possible decomposition of the reflective/control system based on the theoretical assumption that several higher cognitive processes are involved in self-control (Kuhl and Goschke, 1994; Buhringer et al., 2008; Goschke, 2012).

### Correlations between the Task Sub-scores and Conventional Measures of Self-Control Ability

We also analyzed correlations between the sub-scores with previously used tasks that might be considered to measure these sub-components. We expected that distractibility by aversion would correlate with performance on the unsolvable anagram task, that distractibility by temptation would correlate with delay of gratification, and that distractibility by neutral distraction would correlate with Stroop task performance. We could only confirm the second of these hypotheses. That is, participants who chose to eat a tempting sweet directly instead of waiting in order to receive two sweets later also showed a higher distractibility by erotic pictures. More specifically, their gaze distance from the target location was higher. This effect was not present when looking at RT data, indicating that eyetracking data might in some cases be more sensitive in detecting individual differences concerning susceptibility to temptation. Note that the absence of a relation between our temptation RT score and delay decision may also be explained by problems in interpreting the delay task in adults. As has been noted previously, interpretation of the delay task as a measure of self-control can be problematic (Mischel et al., 2011; McGuire and Kable, 2013). However, we designed the task in a manner aimed at reducing confounding effects. Our task measures of aversion did not correlate with the external measure of aversion, the anagram task; and our task measures of distraction did not correlate with the external measure of neutral distraction, the Stroop task. As current questionnaires are not designed to distinguish between aversions, temptations, and neutral distraction, we have no means of determining the relation between task sub-scores and self-report of these facets of self-control. Development of a questionnaire that includes these facets may be conducive to the investigation of individual differences in self-control when having to rely on self-report.

Our results suggest that our measure of susceptibility for temptation (using erotic pictures) generalizes to other types of temptations and situations (i.e., decision-making about food). In contrast, our measure of aversion does not seem to measure the same ability as the unsolvable anagram task. The reason for this might be that unpleasantness induced by disgust is different from unpleasantness induced by a mentally exhausting task. One could also argue that our measure of enduring unpleasantness (aversion) is more pure than the anagram task, because performance on the anagram task also depends on factors other than the ability to endure unpleasantness. For example, we observed that participants who were particularly good with solvable anagrams also kept going longer on the unsolvable anagram, suggesting that continuing on an unsolvable task might depend critically on the experience with such tasks and the expectation of solving it, rather than merely on self-control. This is in line with the idea that self-efficacy beliefs strongly influence self-control ability (Lippke et al., 2009; Schwarzer, 2009). Furthermore, the anagram task might leave some room for strategies and meta-cognitive thoughts of the participants, which might obscure the real correlation between the tasks. Likewise, the validity of our self-control task might also be lowered by the possibility that some participants might have tried to strategically select some trials as “non-control” trials. For example, they might have decided to enjoy the erotic images on some trials, thereby risking not to win the lottery-based bonus payment at the end of the experiment. Assessing risk aversion and an according post-experimental questionnaire on strategic behavior might have provide additional information in this regard.

The absence of a correlation between Stroop task performance and distractibility by neutral pictures seems surprising, as both are attentional paradigms. In the Stroop task, the written word is supposed to distract participants from naming the display color of the text. However, in contrast to our distraction condition, this is an active interference induced by incongruence and not a mere distraction effect. In contrast, our task involves selective spatial attention. Furthermore, Nee et al. (2007) revealed that partially distinct neural mechanisms underlie different sorts of interference resolution tasks including Stroop and Flanker paradigms, suggesting that they might rely on different cognitive mechanisms. Additionally, the Stroop task contains a fairly artificial conflict situation, whereas using photographs as in our task might resemble real-life conflicts more accurately. Finally, the strength of the interference effect of words depends on practice effects (Cohen et al., 1990), which might vary across participants, compromising the extent to which inter-individual differences in the Stroop effect mirror self-control.

A possible future approach may include a more specific sub-categorization of distractor types (e.g., the use of disgusting, sad, and annoying pictures for aversion, and use of erotic, food and emotionally positive pictures for temptation) in order to investigate whether an even higher categorization increases the explanatory power of our task. Furthermore, a future approach may entail the use of real-life self-control conflicts categorized into the different impulse types for validating the task results.

### Eye Gaze Patterns for Aversion, Temptation, and Distraction

Gaze pattern analysis reveals higher gaze distance for temptation and for distraction, but not for aversion as compared to the respective control conditions. Additionally, there was more looking around for all three self-control sub-components, reflected in a higher standard deviation of the gaze distance. This suggests that for temptation and distraction, the pictures attracted overt attention, suggesting that the attentional capture of objects can be an indicator for their attractiveness and that attention allocation forms an important mechanism in self-control behavior. This is in line with the findings that attentional deployment in toddlers is an important factor in predicting the ability to resist short-term temptations later in life (Sethi et al., 2000). Additionally, looking away from the tempting object has also proven to be a useful strategy in Mischel’s delay of gratification experiment (Mischel et al., 1972), indicating that the degree of attention control can be used to predict self-control success.

One possible reason for why we, contrary to our prediction, did not find an effect of aversion on mean gaze distance is that aversion generally might not only trigger a reaction to look away, but also toward the picture, because of its high saliency. Furthermore, instead of looking away, participants might try to regulate their emotions in the face of disgusting pictures. This is in line with the finding that reappraisal forms a common strategy when regulating negative emotions and that this form of emotion regulation has also been found to be more effective in reducing negative affect than pure distraction (McRae et al., 2010). This might explain why aversion elicited different eye gaze correlates as compared to temptation or distraction. Note, that in the current task we cannot completely exclude the possibility that participants could recognize the target letter without fixating it. This could be insured by reducing letter size and presenting a mask after letter presentation in future studies.

## Conclusion

In this work we advance beyond prior approaches to studying self-control by assessing attentional component processes of this trait. We present a self-control task in which participants have to control themselves in the face of aversive events, temptations, and neutral distractions using naturalistic stimuli. Our study indicates that self-control abilities concerning these three impulse categories form independent aspects of self-control.

## Author Contributions

All authors listed, have made substantial, direct and intellectual contribution to the work, and approved it for publication.

## Conflict of Interest Statement

The authors declare that the research was conducted in the absence of any commercial or financial relationships that could be construed as a potential conflict of interest.
