# Supplementary material for: Decomposing Self-Control: Individual Differences in Goal Pursuit Despite Interfering Aversion, Temptation, and Distraction
Source: Front Psychol. 2016 Apr 18;7:382. doi: 10.3389/fpsyg.2016.00382 (PMC4834631; doi:10.3389/fpsyg.2016.00382)
Supplement: Supplementary file 1 [file Appendix.PDF]

Appendix

Appendix A

Exclusion of Participants

One participant was excluded from all analyses because she was diagnosed with social anxiety in another study in our laboratory. Another participant was excluded because of an average accuracy across all conditions of less than 60 percent, indicating that the participant might not have been able to see the target letter at all. Eight participants indicated that they were homosexual. Because we do not know whether or not they differ in their reaction to pictures of heterosexual couples, they were excluded from all data analyses. From another four participants eyetracking data quality was low: more than 30 per cent of samples were missing because the pupil was not detected during this time. These four participants and an additional four for whom no eyetracking data was acquired were excluded from eyetracking data analysis but manual response data were used.

Appendix B
Table of Picture Valence, Arousal, Attraction, Brightness and Complexity
Valence, arousal, and attraction ratings of an independent sample prior to testing. Values on a 9-point Likert-type scale with 1 representing low (valence, arousal, or attraction). Picture brightness and color from white, red, green, blue (255) to black (0), and complexity (entropy in bits) was estimated using the Matlab image processing toolbox.

	Disgusting		neutral ipsilateral		erotic		neutral contralateral	
	M	SD	M	SD	M	SD	M	SD
valence	2.08	0.34	5.09	0.24	7.09	0.21	5.10	0.24
arousal	4.64	0.42	2.84	0.30	6.60	0.30	2.80	0.27
attraction	2.26	0.31	5.01	0.18	6.94	0.21	5.01	0.16
brightness	118.18	24.02	126.07	30.71	106.82	36.87	118.01	40.16
entropy	7.53	0.40	7.39	0.47	7.39	0.52	7.21	0.53
red	132.75	32.07	136.55	32.42	133.12	38.81	126.78	39.17
green	113.71	24.20	124.23	32.77	98.66	37.21	116.35	41.85
blue	103.00	24.84	108.07	39.70	79.88	39.71	103.59	46.96

Appendix C

Delay of gratification questionnaire - German version used in the study

1. Wie lange ist es her, dass Sie etwas gegessen haben? (in Stunden) _______

2. Wie hungrig waren Sie nach dem ersten Scandurchlauf?

O O O O O O O

nicht hungrig sehr hungrig

2. Wie gerne mochten Sie die Süßigkeit, die Sie gegessen haben?

O O O O O O O

gar nicht sehr

3. Warum haben Sie sich für die gewählte Option (eine Süßigkeit sofort bzw. zwei Stück später) entschieden?

____

Delay of gratification questionnaire - English translation

1. How long ago did you eat for the last time? (hours) _______

2.How hungry were you after the first scanning session?

O O O O O O O

not hungry very hungry

3. How much did you like the sweets that you have eaten?

O O O O O O O

not at all very much

4. Why did you decide for the option you chose (small sweets immediately vs. two sweets later)?

____

Appendix D

List of anagrams in German

Anagram	Solution
CERKA	ACKER
GNMTAE	MAGNET
LPMSIU	IMPULS
HCTMOHU	HOCHMUT
BRENLEO	-

Appendix E
Table of mean and standard deviation for the different measures per condition
Mean and within subject standard deviation for the percent errors (errors), reaction times in ms (RTs), mean gaze distance (M gaze), standard deviation of the gaze distance (SD gaze), valence ratings, arousal ratings and attraction ratings by the participants in our study.

	disgusting		neutral Ipsilateral		erotic		neutral contralateral		no-distractor	
	M	SD	M	SD	M	SD	M	SD	M	SD
errors	12.21	11.04	10.21	9.56	6.96	7.68	5.66	6.07	4.12	3.99
RTs	655.71	70.54	641.43	69.37	598.17	68.75	591.33	67.57	583.61	60.66
M gaze	3.34	.68	3.29	.57	3.76	.84	3.54	.56	3.32	.41
SD gaze	.41	.22	.35	.20	.55	.40	.40	.21	.18	.24
valence	2.46	.97	5.17	.40	7.03	1.08	5.11	.39	-	-
arousal	4.85	2.05	2.74	1.57	6.55	1.15	2.75	1.54	-	-
attraction	2.41	.917	5.07	.28	6.98	.93	5.00	.28	-	-

Appendix F
Non-Parametric Testing
Non-parametric testing - behavioral data
There was a floor effect in error rates resulting in significantly skewed distributions (Shapiro-Wilk: $p < .001$) for all conditions. When performing a non-parametric Wilcoxon signed-rank test for the error rates, the comparison of erotic with neutral distractors did not hold Bonferroni correction ($p = .017$,. uncorr). The other comparisons remained significant. The mean RTs were normally distributed statistically (Shapiro-Wilk: $p < .10$) and by sight for all conditions.

Non-parametric testing - eyetracking data
Distance data from all five conditions were not normally distributed (Shapiro-Wilk: $p < .05$) for mean gaze distance and standard deviation of gaze distance, with most participants showing a low distance and standard deviation.

The comparison neutral contrast did not survive Bonferroni correction when we applied a non-parametric Wilcoxon signed-rank test ($p = .015$. uncorrected). Additionally, the aversion standard deviation contrast did not survive Bonferroni correction (Wilcoxon signed-rank test: $p = .26$). The other significant comparisons remained significant when non-parametric testing was applied ($p < .05$. Bonferroni corrected for the 6 comparisons).

Appendix G

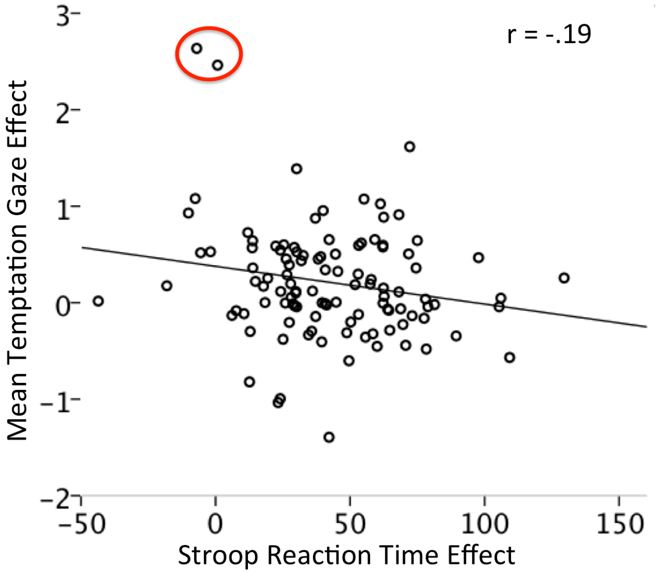

Correlation between the Stroop Reaction Time Effect and the Temptation Gaze Distance Effect. The negative correlation becomes insignificant when the two potential outliers (circled in red) are excluded from analysis.

Appendix H
Table comparing male and female participants
Comparison of reaction times, error rates, mean gaze distance from target location and standard deviation of the gaze distance from target location reveals no significant differences between males and females besides for the standard deviation of the gaze. Note, that the significantly higher standard deviation of gaze distance for male in comparison to female participants does not survive Bonferroni correction for multiple comparisons.

Measure	M Female	M Male	T(df)	p
Arousal	6.52	6.82	-1.42(113)	.158
Valence	7.06	7.39	-1.72(113)	.089
RT	594.61	602.11	-.59(114)	.559
Error	0.06	0.07	-.69(114)	.492
Gaze Distance	3.76	3.75	.014(107)	.989
SD of Gaze Distance	0.41	0.70	-2.43(107)	.017*
